# Transcription factor c-fos induces the development of premature ovarian insufficiency by regulating MALAT1/miR-22-3p/STAT1 network

**DOI:** 10.1186/s13048-023-01212-3

**Published:** 2023-07-21

**Authors:** Ting Qiu, Jie Zhou, Bing Ji, Liuyang Yuan, Tingsong Weng, Huishu Liu

**Affiliations:** 1 Department of Obstetrics and Gynecology, The First Affiliated Hospital of Jinan University, No. 613, West Huangpu Avenue, Tianhe District, Guangzhou, Guangdong Province 510630 P.R. China; 2 Department of Obstetrics and Gynecology, Guangzhou Women and Children’s Medical Center, No. 9, Jinsui Road, Guangzhou, Guangdong Province 510623 P.R. China

**Keywords:** Premature ovarian insufficiency; ceRNA network; c-fos; MALAT1; miR-22-3p; STAT1

## Abstract

**Background:**

The current study attempted to investigate the role of transcription factor c-fos in the development of premature ovarian insufficiency (POI) as well as the underlying mechanism involving the MALAT1/miR-22-3p/STAT1 ceRNA network.

**Methods:**

Bioinformatics analysis was performed to extract POI-related microarray dataset for identifying the target genes. Interaction among c-fos, MALAT1, miR-22-3p, and STAT1 was analyzed. An in vivo POI mouse model was prepared followed by injection of sh-c-fos and sh-STAT1 lentiviruses. Besides, an in vitro POI cell model was constructed to study the regulatory roles of c-fos, MALAT1, miR-22-3p, and STAT1.

**Results:**

c-fos, MALAT1, and STAT1 were highly expressed in ovarian tissues from POI mice and CTX-induced KGN cells, while miR-22-3p was poorly expressed. c-fos targeted MALAT1 and promoted MALAT1 transcription. MALAT1 competitively bound to miR-22-3p and miR-22-3p could suppress STAT1 expression. Mechanically, c-fos aggravated ovarian function impairment in POI mice and inhibited KGN cell proliferation through regulation of the MALAT1/miR-22-3p/STAT1 regulatory network.

**Conclusion:**

Our findings highlighted inducing role of the transcription factor c-fos in POI through modulation of the MALAT1/miR-22-3p/STAT1 ceRNA network.

**Supplementary Information:**

The online version contains supplementary material available at 10.1186/s13048-023-01212-3.

## Background

The ovaries have been found to be a frequent target of the immune system, with approximately 4–30% of cases arising from autoimmune diseases [1]. Premature ovarian insufficiency (POI) is the depletion of ovarian follicles in women under 40 years of age, preventing them from performing the normal work of their reproductive and endocrine organs [1, 2]. POI is characterized by an accelerated decline in ovarian function and early menopause [3]. The etiology of POI is complex and varied, and approximately 90% of cases remain unknown [4].

c-fos is an AP-1 transcription factor that binds to specific enzymes of the endoplasmic reticulum phospholipid synthesis pathway and activates the synthesis of these specific enzymes [5]. c-fos is associated with cancer progression, evidenced by significantly reduced c-fos expression in precancerous lesions in comparison to invasive cervical cancer, and its role in breast cancer as an independent predictor of decreased patient survival [5]. c-fos is a regulator of c-myc-induced cell death and may facilitate apoptosis via the p38 MAP kinase pathway [6]. There is also a relationship between c-fos and ovarian function. It has been demonstrated that c-fos activation increases the expression of genes involved in prostaglandin production and human granulosa lutea cell transport and plays an important role in ovulation [7].

MALAT1 may act as a transcription regulator of many genes, including some engaged in cancer metastasis, and is implicated in the modulation of cell cycle [8]. Elevated MALAT1 expression shows linkage with poor overall survival in various types of cancer, highlighting its critical prognostic role in various cancers [9]. MALAT1 has been shown to be transcriptionally activated by c-fos and regulates downstream miRNAs [10]. Bioinformatics analysis performed in the present study predicted miR-22-3p as the differentially expressed miRNA in POI with the most significant change. It was found that MALAT1 targets miR-22-3p through inhibition of the PI3K/Akt pathway, thereby affecting the proliferation and migration of kidney cancer cells [11]. It was also reported that high expression of MALAT1 can targetedly downregulate miR-22-3p, thereby inhibiting proliferation and inducing apoptosis in gastric cancer cells [12]. In addition, miR-22-3p is expressed at low levels in POI, and reduced miR-22-3p in POI plasma may reflect reduced ovarian reserve in patients as a result of the pathological process of POI [13]. Of note, upregulation of MALAT expression could regulate the activation of STAT1 signaling, which promotes the production of inflammatory cytokines and MMP [14].

Herein, we explored the possible molecular mechanisms involved in the involvement of the transcription factor c-fos in regulating the MALAT1/miR-22-3p/STAT1 ceRNA network in the development of POI, and our study may provide a new theoretical basis for the diagnosis and treatment of POI.

## Methods

### GEO microarray data analysis

Utilizing the GEO database, the POI related miRNA expression microarray GSE100238 (10 *vs.* 10 granulosa cell microarray sample dataset) was retrieved, including granulosa cell samples from 10 patients with biological premature ovarian efficiency (bPOI) and 10 age- and BMI-matched normal control women. The R language "limma" package was used to screen the POI related genes, with |logFC|> 1 and *p* value < 0.05 set as the threshold values, and the different analysis was carried out on the granulosa cells and bPOI samples of normal control women, followed by display of the heatmap of 20 miRNAs with significant changes. Through the StarBase database, the binding sites between the miRNA and gene and between the miRNA and lncRNA were predicted, respectively. The HTFtarget database was used to verify whether there is a targeting relationship between the lncRNA and upstream transcription factors.

### In vivo POI mouse model construction

Sixty SPF-grade female C57BL/6N mice (6 ~ 8 weeks old) were purchased from the Shanghai Experimental Animal Centre. All mice were randomly housed in cages and kept under standard laboratory conditions, with a 12-h light/dark cycle (light on at 7 am), 60% humidity, and free access to water and food at 24 °C. The experimental animals were randomly used as normal controls or for POI modeling without additional treatment or further injected with lentiviral vectors expressing short hairpin RNA (sh)-negative control (NC) + overexpression (oe)-NC, sh-c-fos + oe-NC, or sh-c-fos + oe-STAT1 via tail vein (*n* = 12). The specific process of POI mouse model construction is shown in Figure S1. A single intraperitoneal injection of cyclophosphamide (CTX, Sigma-Aldrich, St. Louis, MO, 120 mg/kg) and busulfan (BUS, Sigma-Aldrich, 12 mg/kg) was used to establish the POI mouse model. The control mice were injected with equal amounts of PBS. After 2 weeks [15], lentiviral vectors expressing sh-NC, sh-c-fos and sh-STAT1 (300 μL, 1.5 × 10^8^ TU/mL, GenePharma, Shanghai, China) was administered to the POI model mice via the tail vein every 2 days for 2 weeks. This experiment was approved by the animal ethics committee of our hospital.

### In vitro POI cell model construction

The human ovarian granulosa-like tumour cell line KGN was purchased from Feiya Biotechnology Ltd. KGN was cultured in DMEM/F12 medium (10,569,044, Gibco, Carlsbad, CA) appended to 10% FBS (10,099,141, Gibco), penicillin (100 U/mL) and streptomycin (100 U/mL) (15,070,063, Gibco) in a cell incubator at 37 °C with 5% CO_2_. KGN cells were cultured with 250 μM CTX for 48 h to construct an in vitro POI cell model [15, 16].

### Cell treatment

The overexpression plasmids and shRNAs and corresponding NCs were purchased from Genechem (Shanghai, China). Plasmids were transfected into cells utilizing Lipofectamine 3000™ (Life Technologies, Carlsbad, CA). Cell transfection was performed for 48 h after successful construction of the POI cell model (48 h after CTX induction) [17–19]. Transfections were grouped as following.

KGN cells after CTX treatment were further treated with sh-NC, sh-c-fos, oe-NC, oe-c-fos, oe-MALAT1, sh-MALAT1, inhibitor-NC, mimic-NC, miR-22-3p inhibitor, miR-22-3p mimic, sh-STAT1, or oe-STAT1. The gene silencing sequences were designed as follows: sh-NC (CCACCGTCACACACAGTATTTAT), sh-MALAT1 (1: CCTCAGACAGGT ATCTCTT; 2. GATCCATAATCGGTTTCAA), sh-c-fos (1: GGTGGAACAGTTATCTCCAGAAGAA; 2: CCGGGACACACCCTTACTCTCCAAAC).

### Enzyme-linked immunosorbent assay (ELISA)

Blood was collected at the time of sacrifice of the mice, left at ambient temperature for 30 min and then centrifuged at 2500 rpm/min for 15 min to obtain serum. Anti-Mullerian hormone (AMH), E2 and FSH levels were tested utilizing ELISA kits (Mlbio, Shanghai, China). The absorbance values were detected at 450 nm and the concentrations were calculated based on standards [20].

### Histological staining and follicle count

Ovaries were fixed overnight in 4% paraformaldehyde, dehydrated and paraffin-embedded, and serially sectioned. Paraffin Sects. (5 μm thick) were stained with hematoxylin and eosin (H&E). Primordial, primary, secondary and mature ovarian follicles were observed and counted using a microscope (BX63, Olympus, Tokyo, Japan). The number of follicles was counted at different locations in the sections and the classification and characteristics of the various stages of follicular development were determined as previously published [15, 21].

### Immunofluorescence staining

Paraffin specimens were dewaxed, incubated in 3% H_2_O_2_ (diluted in methanol) for 10 min at room temperature, sealed with 5% BSA (dissolved in PBS), and incubated for 15 min at room temperature. Paraffin specimens were then incubated with primary antibody rabbit anti-cleaved caspase-3 (ab32042, 1:300, Abcam, Cambridge, UK) overnight at 4 °C and with HRP-labeled secondary antibody goat anti-rabbit polyclonal IgG (1:500, ab6721, Abcam) for 1 h at 37 °C. Afterwards, paraffin specimens were colorized with DBA, counterstained with hematoxylin, sealed with neutral gum, and observed by a microscopy [22].

### RT-qPCR

Total tissue or cellular RNA was extracted using Trizol (16,096,020, Thermo Fisher Scientific, Rockford, IL). miRNA and mRNA were reversely transcribed using the TaqMan™ MicroRNA Reverse Transcription Kit (4,366,596, Thermo Fisher Scientific) and the High-Capacity cDNA Reverse Transcription Kit (4,368,813, Thermo Fisher Scientific), respectively. The primer sequences were designed and provided by Sangon Biotech (Shanghai, China), as shown in Table S1. U6 was used as the internal reference for miR-22-3p and GAPDH for c-fos, MALAT1 and STAT1. RT-qPCR was completed with the RT-qPCR kit (117,320, Thermo Fisher Scientific). The 2^−ΔΔCt^ was adopted for gene expression analysis.

### Western blot

Total protein extracts were subjected to electrophoretic separation on 8%-12% SDS gels and transferred to a PVDF membrane (1,620,177, Bio-Rad Laboratories, Hercules, CA). Next, 5% skimmed milk or 5% BSA was used for blocking the proteins for 1 h at ambient temperature. Primary antibodies were added to incubate the membrane overnight at 4 °C (Table S2). HRP-labeled goat anti-rabbit IgG (ab6721, 1:5000, Abcam) was added dropwise for incubating the membrane at ambient temperature for 1 h. The membrane was immersed in ECL reaction solution (#1,705,062, Bio-Rad) for 1 min, and the protein bands were imaged on an Image Quant LAS 4000C gel imager (GE Healthcare, Milwaukee, WI).

### CCK-8 assay

Cell proliferation assays were carried out with the help of cell counting kit-8 (CCK-8) kits (C0037, Beyotime, Shanghai, China). Subsequently, 10 μL of CCK-8 reagent was added to each well after different cell treatments for 0 h, 24 h, 48 h and 72 h. The cells were incubated in the incubator for 1 h. The absorbance values were detected at 450 nm utilizing a multifunctional Microplate reader Varioskan LUX (VL0L00D0, Thermo Fisher Scientific) and the cell proliferation curve was plotted [23].

### EdU assay

Cells were seeded in 24-well plates, and EdU was added to the culture medium to reach a concentration of 10 µmol/L, followed by 2-h of incubation. The cells were fixed in PBS solution containing 4% paraformaldehyde for 15 min at room temperature, incubated for 20 min at room temperature with 0.5% Triton-100-containing PBS, and then treated with 100 µL Apollo® 567 (CA1170, Solarbio, Beijing, China) at room temperature in darkness for 30 min. Cell nuclei were stained with DAPI for 5 min, after which the number of positive cells in each field of view was checked under a fluorescence microscope (FM-600, Puda Optical Chemical Instrument Co. Ltd., Shanghai, China) [24].

### RNA immunoprecipitation (RIP)

MALAT1 binding to miR-22-3p protein was detected using the RIP kit (17–701, Millipore, Billerica, MA). Antibodies used for RIP were: rabbit anti-mouse AGO2 (1:100, ab32381, Abcam) and goat anti-mouse IgG (1:100, ab205719, Abcam, NC) [25].

### RNA pull-down assay

Incubation of biotin-labeled Bio-NC, Bio-MALAT1-WT, and Bio-MALAT1-MUT RNA (50 nM each) with cells was performed. After 48 h, cells were then incubated with specific cell lysate for 10 min, after which 50 mL of sample cell lysate was dispensed and the residual lysate was incubated for 3 h at 4 °C with M-280 streptavidin magnetic beads pre-coated with RNase-free and yeast tRNA. The bound RNA was purified by Trizol, followed by RT-qPCR to detect enrichment of miR-22-3p.

### Dual-luciferase assay

The hTFtarget database was utilized for prediction of upstream regulatory transcription factors of MALAT1. The Jaspar, StarBase and TargetScan databases were applied for predicting the target binding sites between c-fos and MALAT1, between miR-22-3p and MALAT1, and between miR-22-3p and STAT1, respectively; target binding sites existed both in human and mice.

MALAT1 and STAT1 gene fragments (MALAT1-WT and STAT1-WT) containing the predicted binding sites were synthesized and introduced with mutated fragments (MALAT1-MUT and STAT1-MUT) into the pGL3-basic vector (E1751, Promega, Madison, WI). Following restriction endonuclease, the target fragments were inserted into the pGL3 vector using T4 DNA ligase. The Renilla luciferase reporter plasmid and the constructed firefly luciferase reporter plasmid were co-transfected with mimic-NC and miR-22-3p mimic into 293 T cells, respectively. The luciferase activity was assayed utilizing the Dual-Luciferase® Reporter Assay System kit (E1910, Promega) on a GloMax® 20/20 Luminometer (E5311, Promega). All vectors were prepared by Sangon Biotech (Table S3).

### Chromatin immunoprecipitation (ChIP)

ChIP assays were performed using the EZ-Magna ChIP kit (17–10,461, Sigma-Aldrich) with antibodies: goat anti-mouse IgG (ab205719, 1:100, Abcam, NC) and c-fos antibody (ab27793, 1:20, Abcam). Finally, the amount of precipitated MALAT1 was analysed by RT-qPCR.

### Flow cytometry

Cells (5 × 10^5^) were detached with EDTA-free trypsin and centrifuged at 300 g for 5 min at 4 °C. Cell precipitates were washed twice in pre-cooled PBS. According to the instructions of BIomake Apoptosis Kit (B32117, Houston, TX), 100 µL of binding buffer was added to resuspend the cells, followed by cell incubation with 5 µL of V-FITC and 5 µL of PI staining solution for 10 min at room temperature. Next, 400 µL binding buffer and mixture were added to the cells and the apoptosis rate was tested by flow cytometry; the sum of PI^−^ AnnexinV^+^ (early apoptotic cells) and PI^+^ AnnexinV^+^ (late apoptotic cells / dead cells) represented apoptotic cells [26].

### TUNEL staining

Cells were fixed in PBS containing 4% formaldehyde on a coverslip for 30 min, perforated with Triton X-100 (1%) for 5 min and then infiltrated in 3% hydrogen peroxide for 10 min. Cells were incubated for 1 h at 37 °C with TdT labeling solution (C1086, Beyotime). The washed cells were incubated with 100 μL staining buffer solution for 30 min (dark condition) and stained with DAPI [15, 27].

### Statistical analysis

All data were processed utilizing SPSS 21.0 statistical software (IBM Corp. Armonk, NY), and measurement data were summarized as mean ± standard deviation. Independent sample t-test was adopted for comparison between two groups, and one-way ANOVA for comparison between multiple groups, followed by Tukey's post-hoc test. *p* < 0.05 indicated statistically significant difference.

## Results

### c-fos and MALAT1 are highly expressed in both in vivo mouse models and in vitro cell models of POI

**Fig. 1:**
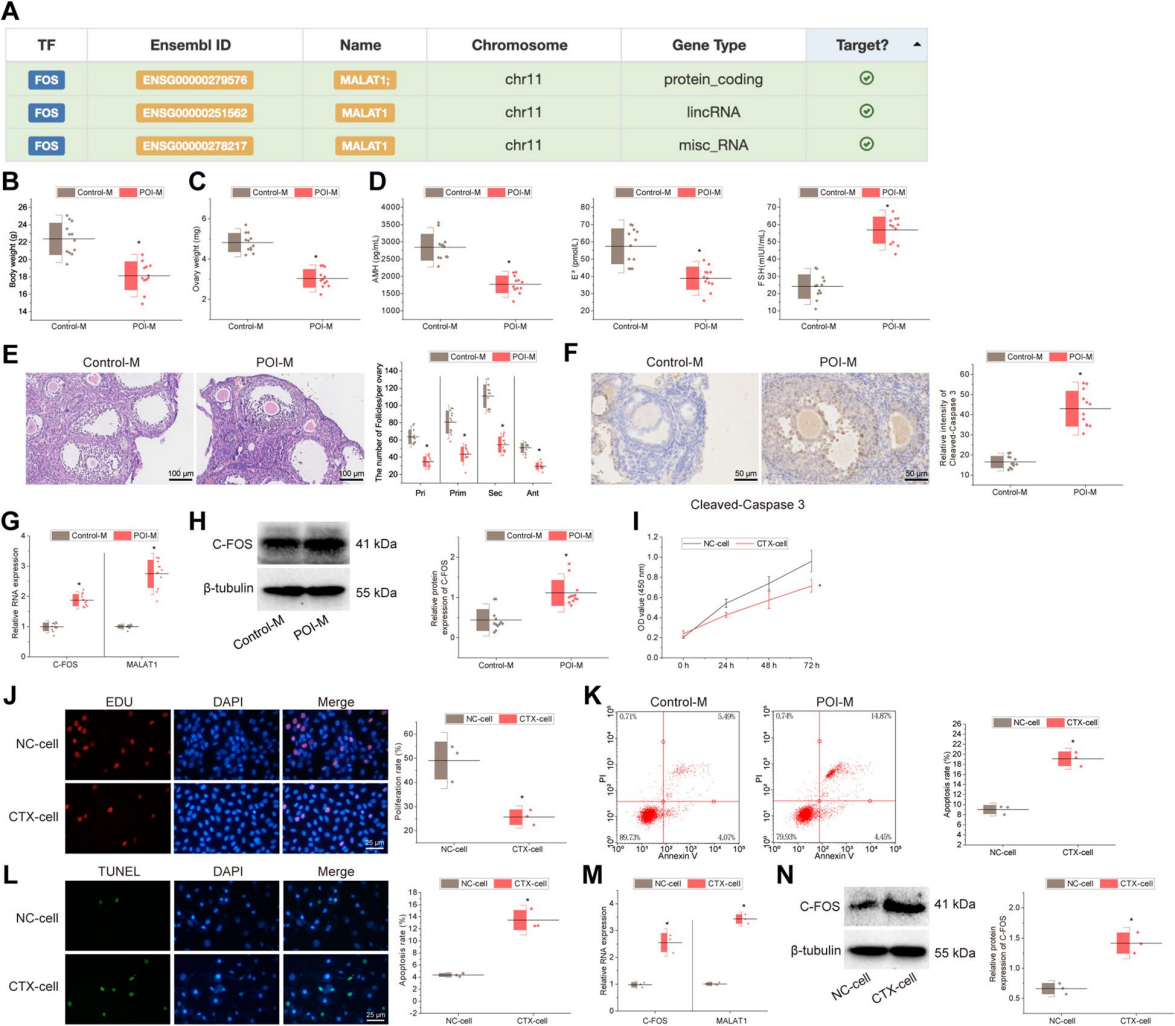
Expression of c-fos and MALAT1 in in vivo mouse models and in vitro cell models of POI. **A** The targeting relationship of MALAT1 with transcription factor c-fos obtained from the hTFtarget database; **B** Body weight of mice measured 14 days after CTX and BUS induction. *n* = 12. * *p* < 0.05 *vs.* control mice; **C** The ovarian weight of mice measured 14 days after CTX and BUS induction. *n* = 12. * *p* < 0.05 *vs.* control mice; **D** ELISA for serum AMH, E2 and FSH levels in mice. * *p* < 0.05 *vs.* control mice; **E** H&E staining for ovarian tissue structure in mice and statistical results of the number of follicles in each ovary. *n* = 12. * *p* < 0.05 *vs.* control mice; **F** Cleaved-Caspase3 immunohistochemistry to detect apoptosis in ovaries of mice and statistical results of relative intensity of positive staining. *n* = 12. * *p* < 0.05 *vs.* control mice; **G** RT-qPCR to detect the expression of MALAT1 and c-fos in ovarian tissues of mice. * *p* < 0.05 *vs.* control mice; **H** Western blot to detect the protein expression of c-fos in ovarian tissues of mice and the statistical plot of the gray values of the protein bands. * *p* < 0.05 *vs.* control mice; **I** Cell viability detected by CCK-8. * *p* < 0.05 *vs.* NC cells; **J** Cell proliferation detected by EdU assay and statistical results of proliferation rate. * *p* < 0.05 *vs.* NC cells; **K** Cell apoptosis detected by flow cytometry and statistical results of apoptosis rate. * *p* < 0.05 *vs.* NC cells. **L** TUNEL staining for apoptosis detection and statistical results of apoptosis rate. * *p* < 0.05 *vs.* NC cells; **M** RT-qPCR for MALAT1 and c-fos expression. * *p* < 0.05 *vs.* NC cells; **N** Western blot for c-fos protein expression and the statistical plot of the gray values of the protein bands. * *p* < 0.05 *vs.* NC cells. Cell experiments were repeated three times

It has been documented that c-fos is highly expressed in POI samples [28]. MALAT1 gene may be involved in the regulation of AMH signaling defects in POI patients [29]. In our study, we used the hTFtarget database for prediction of the upstream regulatory transcription factor of MALAT1, and found that c-fos had a target binding relationship with MALAT1 (Fig. 1A). Furthermore, c-fos has been shown to promote MALAT1 expression [10]. Therefore, we hypothesized that c-fos may regulate MALAT1 expression and participate in the regulation of the pathogenesis of POI.

An in vivo model of POI was constructed by intraperitoneal injection of CTX and BUS into mice. The POI mice showed a significant decrease in both body weight and ovarian weight on day 14 after induction (Fig. 1B-C). In addition, ELISA results showed that AMH and E2 levels were lower in the POI mice than in the control mice, while FSH was higher (Fig. 1D). H&E staining displayed that the numbers of primordial, primary, secondary follicles, and antral follicles were reduced in the POI mice, with irregular granulosa cell layers and increased inflammatory cell infiltration (Fig. 1E). Cleaved-Caspase3 immunohistochemical analysis displayed more apoptotic ovarian granulosa cells in the POI mice than in the control mice (Fig. 1F). Altogether, the POI mouse model was successfully constructed.

RT-qPCR and Western blot assays depicted that MALAT1 expression was increased in the ovarian tissue of the POI mice compared to the control mice, and c-fos mRNA and protein expression was increased (Fig. 1G-H). KGN cells were treated with 250 μM CTX for 48 h to construct an in vitro POI cell model. From CCK-8 and EdU assay results, KGN cells showed a significant decrease in cell viability and proliferation after CTX induction (Fig. 1I-J). Flow cytometry and TUNEL staining results revealed that apoptosis was markedly increased in KGN cells after CTX induction (Fig. 1K-L). These results indicated that the CTX-treated POI cell model was successfully constructed.

Furthermore, both MALAT1 and c-fos expression was increased in the CTX-treated cells than in NC cells (Fig. 1M-N).

### The transcription factor c-fos targets and binds to MALAT1 and promotes MALAT1 transcription

**Fig. 2:**
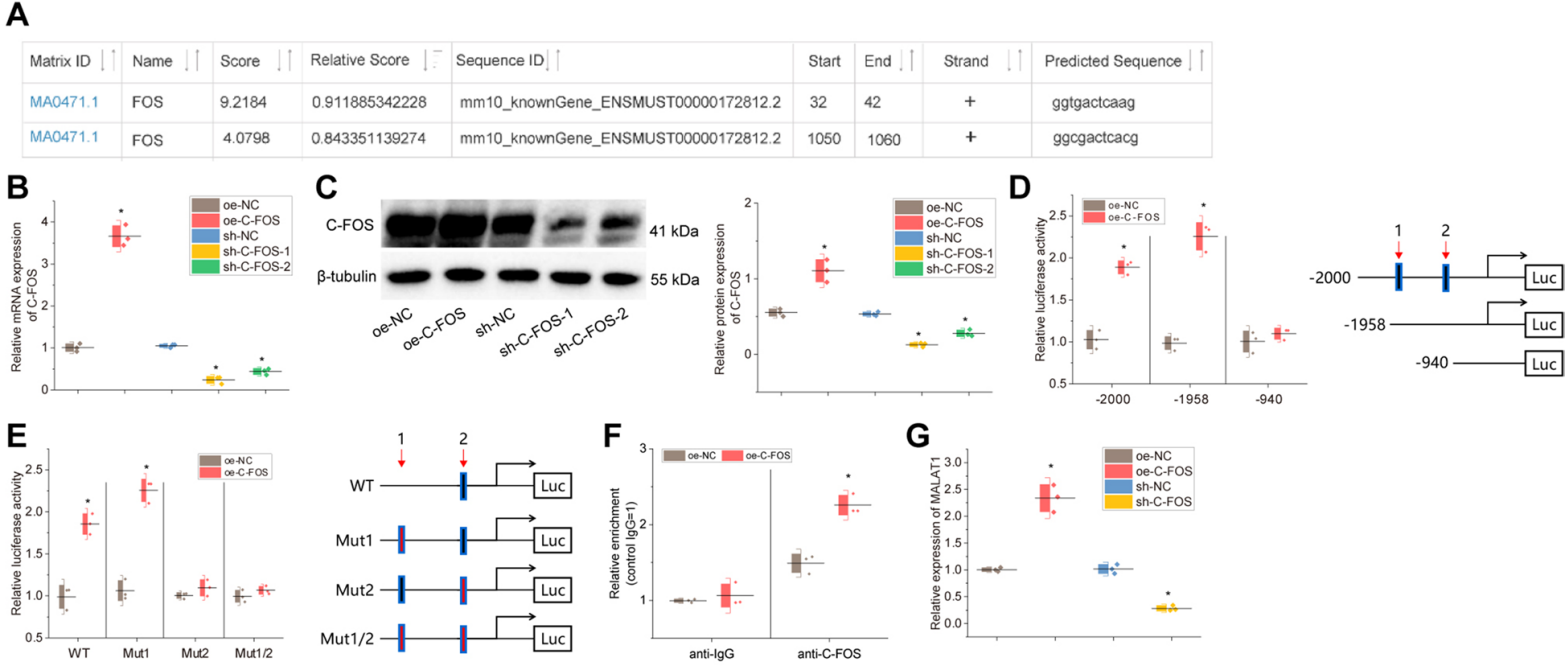
Validation of the regulatory mechanism of c-fos on MALAT1. **A** The JASPAR-database-based online prediction analysis of c-fos binding sites in the promoter region of MALAT1; **B** RT-qPCR detection of the efficiency of interfering and overexpressing c-fos and the statistical plot of the gray values of the protein bands. * *p* < 0.05 *vs.* cells treated with oe-NC or sh-NC; **C** Western blot detection of the efficiency of interfering and overexpressing c-fos and the statistical plot of the gray values of the protein bands; **D**, **E** Dual-luciferase reporter assay to detect the effect of c-fos on MALAT1 viability. * *p* < 0.05 *vs.* cells treated with oe-NC; **F** ChIP assay to detect the binding of c-fos to MALAT1 promoter. * *p* < 0.05 *vs.* cells treated with oe-NC; **G** RT-qPCR to detect MALAT1 expression after knockdown or overexpression of c-fos. * *p* < 0.05 *vs.* cells treated with oe-NC or sh-NC. Cell experiments were repeated three times

We further validated the regulatory relationship between c-fos and MALAT1. We found that c-fos had binding sites in the promoter region of MALAT1 through the JASPAR database (Fig. 2A). Next, we overexpressed or knocked down c-fos in KGN cells, and found that in the CTX-treated cells, c-fos expression was notably increased in the presence of oe-c-fos treatment and diminished in response to sh-c-fos-1 or sh-c-fos-2 treatment (Fig. 2B-C). Since the interference efficiency of sh-c-fos-1 was slightly better than that of sh-c-fos-2, sh-c-fos-1 was selected for the follow-up experimentations. In addition, knockdown of c-fos could promote proliferation and inhibit apoptosis of KGN cells, while overexpression of c-fos had the opposite effect (Figure S2A-F), consistent with the effect of overexpression of c-fos in POI mice.

The results of the dual-luciferase assay revealed that the ability of the oe-c-fos group to activate MALAT1 was markedly reduced when site 2 was truncated or mutated, whereas truncation of site 1 did not affect the ability of oe-c-fos to activate MALAT1, demonstrating that site 2 was the specific site where the oe-c-fos transcription factor bound to MALAT1 DNA (Fig. 2D-E). ChIP assay further verified that c-fos could bind to the MALAT1 promoter (Fig. 2F). RT-qPCR results showed that in the CTX-treated cells, the expression of MALAT1 was increased by c-fos overexpression but reduced by c-fos knockdown (Fig. 2G).

### c-fos inhibits KGN cell proliferation and promotes apoptosis by promoting MALAT1 expression

**Fig. 3:**
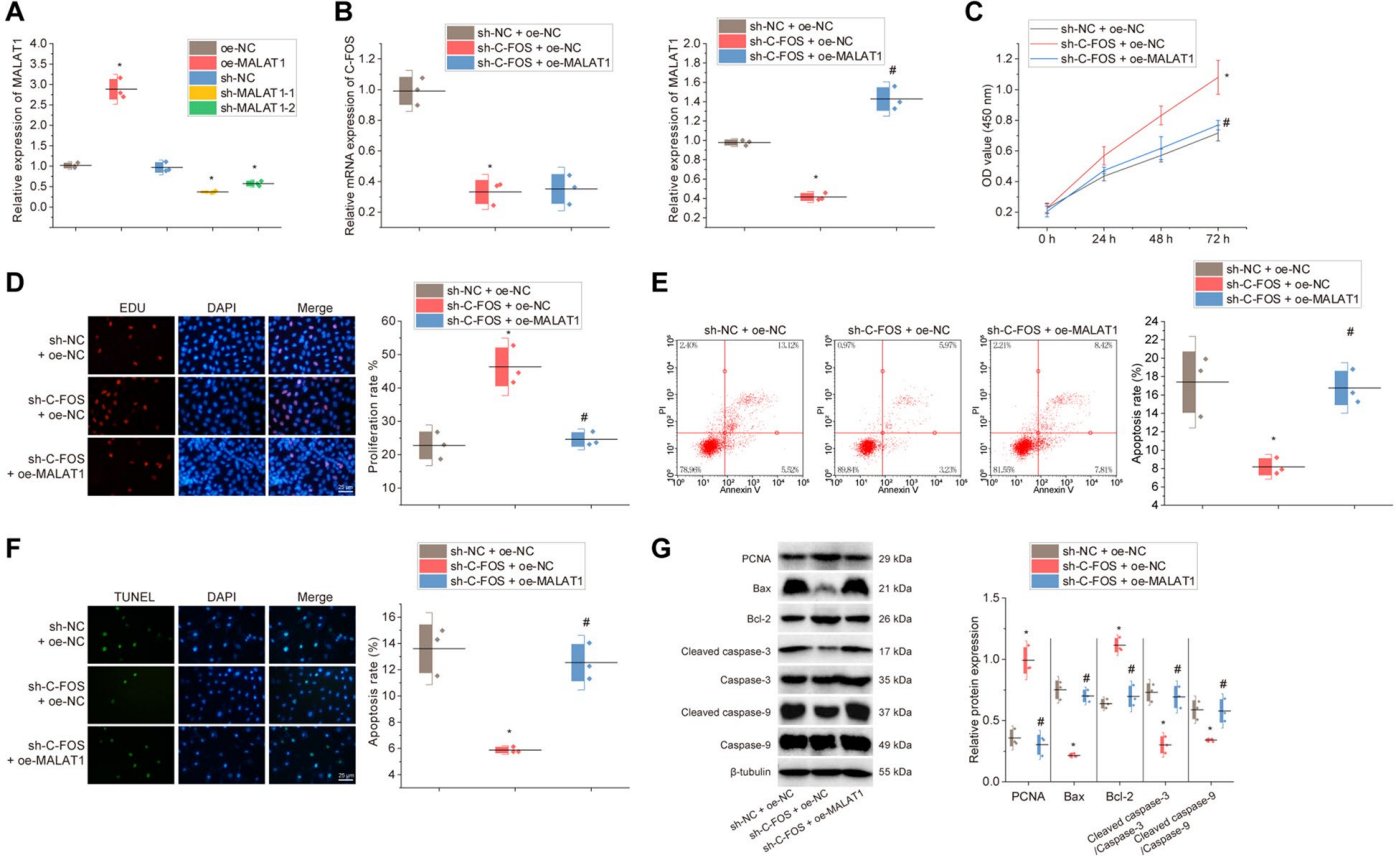
Effects of c-fos on apoptosis and proliferation of KGN cells through regulation of MALAT1 expression. **A** RT-qPCR to detect the efficiency of overexpressing and interfering MALAT1 expression. * *p* < 0.05 *vs.* cells treated with oe-NC or sh-NC. KGN cells were treated with sh-c-fos alone or combined with oe-MALAT1. **B** RT-qPCR or Western blot to detect the expression of MALAT1 and c-fos in cells and gray scale scanning of the protein bands; **C** CCK-8 assay to detect cell viability; **D** EdU assay to detect cell proliferation and statistical results of proliferation rate; **E** Flow cytometry to detect apoptosis and statistical results of apoptosis rate; **F** TUNEL staining to detect apoptosis and statistical results of apoptosis rate; **G** Western blot to detect the expression of proteins related to cell proliferation and apoptosis and statistical results of scale values of the protein bands. In Panels **B**-**G**, * *p* < 0.05 *vs.* cells treated with sh-NC + oe-NC; # *p* < 0.05 *vs.* cells treated with sh-c-fos + oe-NC. Cell experiments were repeated three times

To confirm whether c-fos induced POI by promoting MALAT1 expression, we performed RT-qPCR on KGN cells with different treatments. oe-MALAT1 treatment contributed to higher MALAT1 expression. sh-MALAT1-1 or sh-MALAT1-2 resulted in decreased MALAT1 expression, and sh-MALAT1-1 with optimal interference efficiency was selected for the follow-up experiment (Fig. 3A).

Next, we examined the regulatory effect of c-fos on MALAT1. RT-qPCR results showed that in the CTX-treated KGN cells, c-fos and MALAT1 expression was reduced by sh-c-fos treatment, while further treatment with oe-MALAT1 resulted in no significant difference in c-fos expression but a marked increase in MALAT1 expression (Fig. 3B). Functional assays depicted that the cell viability and proliferation of the CTX-treated KGN cells were notably increased by c-fos knockdown, which could be reversed by MALAT1 overexpression (Fig. 3C-D).

In addition, in the KGN cells after CTX treatment, apoptosis was reduced in the presence of sh-c-fos, while additional MALAT1 overexpression could counteract the effect (Fig. 3E-F). Western blot results showed that in the CTX-treated KGN cells, the expression of the proliferation marker protein PCNA was increased and that of the apoptosis marker proteins Bax, Cleaved caspase-3 and Cleaved caspase-9 was decreased in response to c-fos knockdown, accompanied by elevated Bcl-2 expression; the effects were reversed by MALAT1 overexpression (Fig. 3G).

### MALAT1 can act as a ceRNA to competitively bind to miR-22-3p

**Fig. 4:**
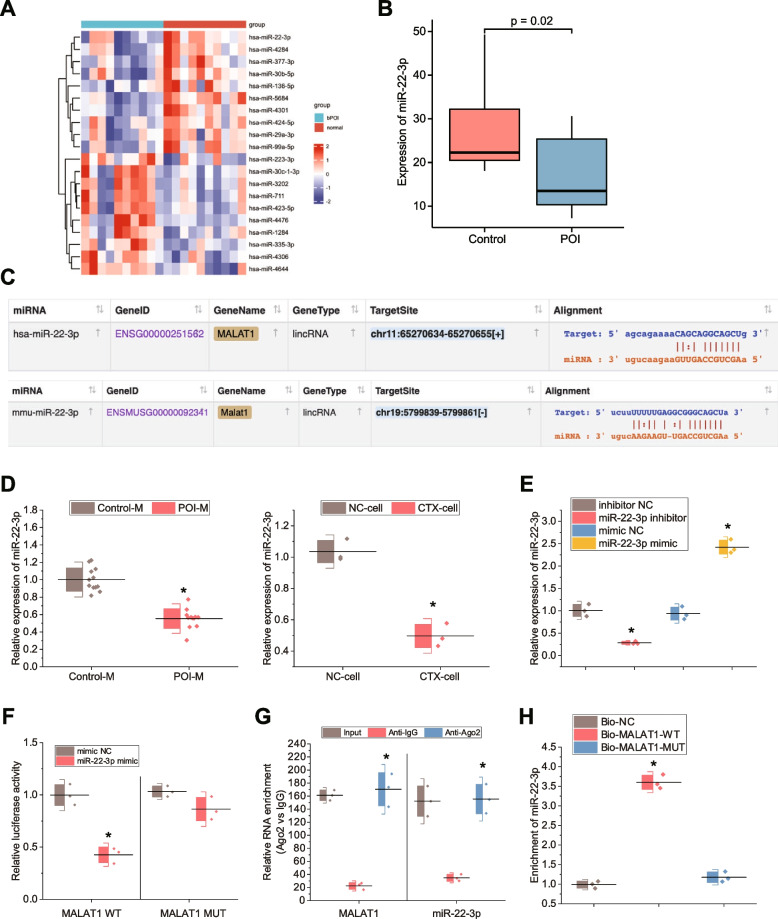
Regulation of miR-22-3p expression by MALAT1 as a ceRNA. **A** Heatmap of differential expression of the top 20 miRNAs in terms of expression change in the bPOI-related miRNA expression microarray GSE100238 in the GEO database, where the color scale from blue to red indicates low to high expression; **B** Box plot of miR-22-3p expression in bPOI samples and control samples (bPOI: *n* = 10, Control: *n* = 10); **C** The target binding site of MALAT1 with miR-22-3p observed by the StarBase database; **D** RT-qPCR to detect miR-22-3p expression in animal and cell models. *n* = 12, * *p* < 0.05 *vs.* control mice or NC cells; **E** RT-qPCR to detect the efficiency of overexpressing and interfering miR-22-3p. * *p* < 0.05 *vs.* inhibitor-NC group or mimic-NC group; **F** Dual-luciferase reporter assay to detect targeted binding of MALAT1 to miR-22-3p. * *p* < 0.05 *vs.* mimic-NC group; **G** RIP assay to detect binding of MALAT1 to miR-22-3p. * *p* < 0.05 *vs.* anti-IgG group; **H** RNA pull-down assay to detect MALAT1 binding to miR-22-3p. * *p* < 0.05 *vs.* Bio-NC group. Cell experiments were repeated three times

To further explore the downstream miRNAs of MALAT1, we differentially analyzed 10 bPOI and 10 granulosa cell samples from normal control females from the POI-related miRNA expression microarray GSE100238 in the GEO database. Accordingly, top 20 differentially expressed miRNAs in terms of expression changes were obtained (Fig. 4A-B), among which miR-22-3p had the highest fold change (Fig. 4B).

It has been documented that miR-22-3p is poorly expressed in human POI, and inhibits human POI deterioration [13]. The StarBase database predicted that MALAT1 and miR-22-3p had target binding sites in mice (Fig. 4C). RT-qPCR depicted that miR-22-3p expression was notably lower in the POI mice compared to the control mice and that miR-22-3p expression was lower in the CTX-treated cells compared to the NC cells (Fig. 4D). These results suggest that miR-22-3p is poorly expressed in POI.

We further determined the targeting relationship between MALAT1 and miR-22-3p. RT-qPCR results showed that miR-22-3p expression was decreased in the presence of miR-22-3p inhibitor but increased in the presence of miR-22-3p mimic (Fig. 4E). Luciferase assay revealed that miR-22-3p mimic inhibited luciferase activity in the MALAT1-WT group, while it had no significant effect on the luciferase activity in the MALAT1-MUT group (Fig. 4F).

We further validated the binding between MALAT1 and miR-22-3p using RIP assays and showed that the amounts of both miR-22-3p and MALAT1 pulled down by AGO2 were higher than that of IgG (Fig. 4G). For the RNA pull-down assay, we transfected the biotin-labeled MALAT1 3'UTR into cells. RT-qPCR detection demonstrated that the miR-22-3p expression was markedly increased in the Bio-MALAT1-WT group but had no significant difference in the Bio-MALAT1-MUT group (Fig. 4H).

### MiR-22-3p targets and inhibits STAT1

**Fig. 5:**
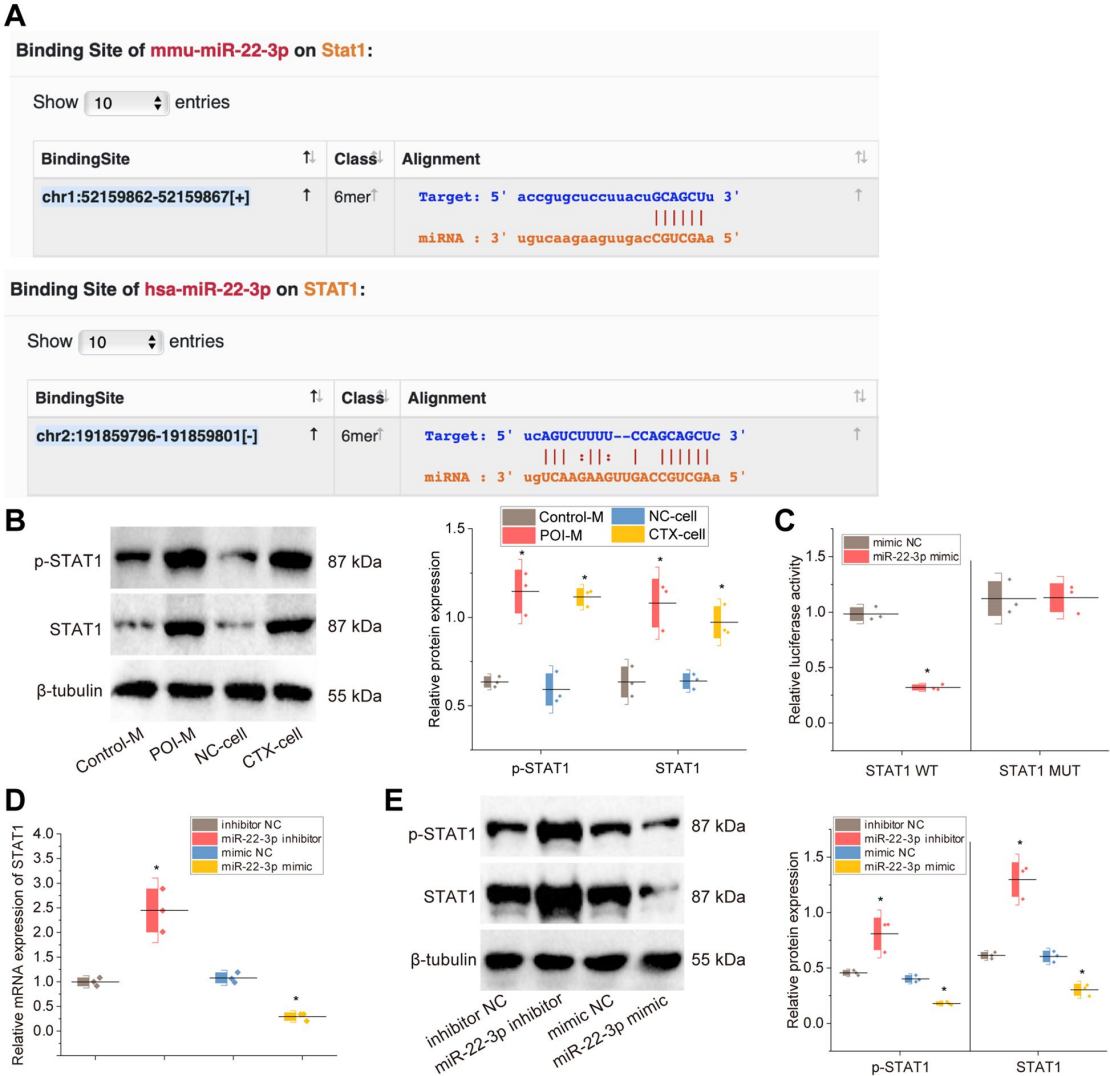
Validation of the targeting and regulation relationship between miR-22-3p and STAT1. **A** The target binding site of STAT1 with miR-22-3p found through the StarBase database. The up arrows indicate the existence of the binding site in human and down arrows indicate that in mice; **B** Western blot detection of STAT1 protein expression and phosphorylation level in mice and cell models and statistical results of scale values of the protein bands. *n* = 12, * *p* < 0.05 *vs.* control mice or NC cells; **C** Dual-luciferase reporter assay to detect the target binding site of STAT1 to miR-22-3p. * *p* < 0.05 *vs.* mimic-NC group; **D** RT-qPCR to detect STAT1 expression in cells. * *p* < 0.05 *vs.* the inhibitor-NC group or mimic-NC group; **E** Western blot to detect STAT1 protein expression and phosphorylation level in cells and statistical results of scale values of the protein bands. * *p* < 0.05 *vs.* inhibitor-NC group or mimic-NC group. Cell experiments were repeated three times

Further, as predicted by the StarBase database, STAT1 and miR-22-3p had target binding sites in both human and mice (Fig. 5A). Western blot results displayed that both STAT1 protein expression and STAT1 phosphorylation level were notably higher in the POI mice than in the control mice (Fig. 5B). In in vitro experiments, both STAT1 protein expression and STAT1 phosphorylation level were increased in the CTX-treated cells compared to the NC cells (Fig. 5B). These results suggest that STAT1 protein expression and STAT1 phosphorylation level are high in POI.

Since STAT1 and miR-22-3p had opposite expression trends in POI, we further verified the targeting relationship between STAT1 and miR-22-3p using a dual-luciferase assay. The results demonstrated that miR-22-3p mimic was able to inhibit the luciferase activity of the STAT1-WT group, while it had no significant effect on the luciferase activity of the STAT1-MUT group (Fig. 5C). Besides, miR-22-3p inhibitor increased STAT1 expression in KGN cells after CTX treatment, while miR-22-3p mimic decreased it (Fig. 5D-E).

### c-fos inhibits proliferation and promotes apoptosis of KGN cells by regulating the MALAT1/miR-22-3p/STAT1 ceRNA network

**Fig. 6:**
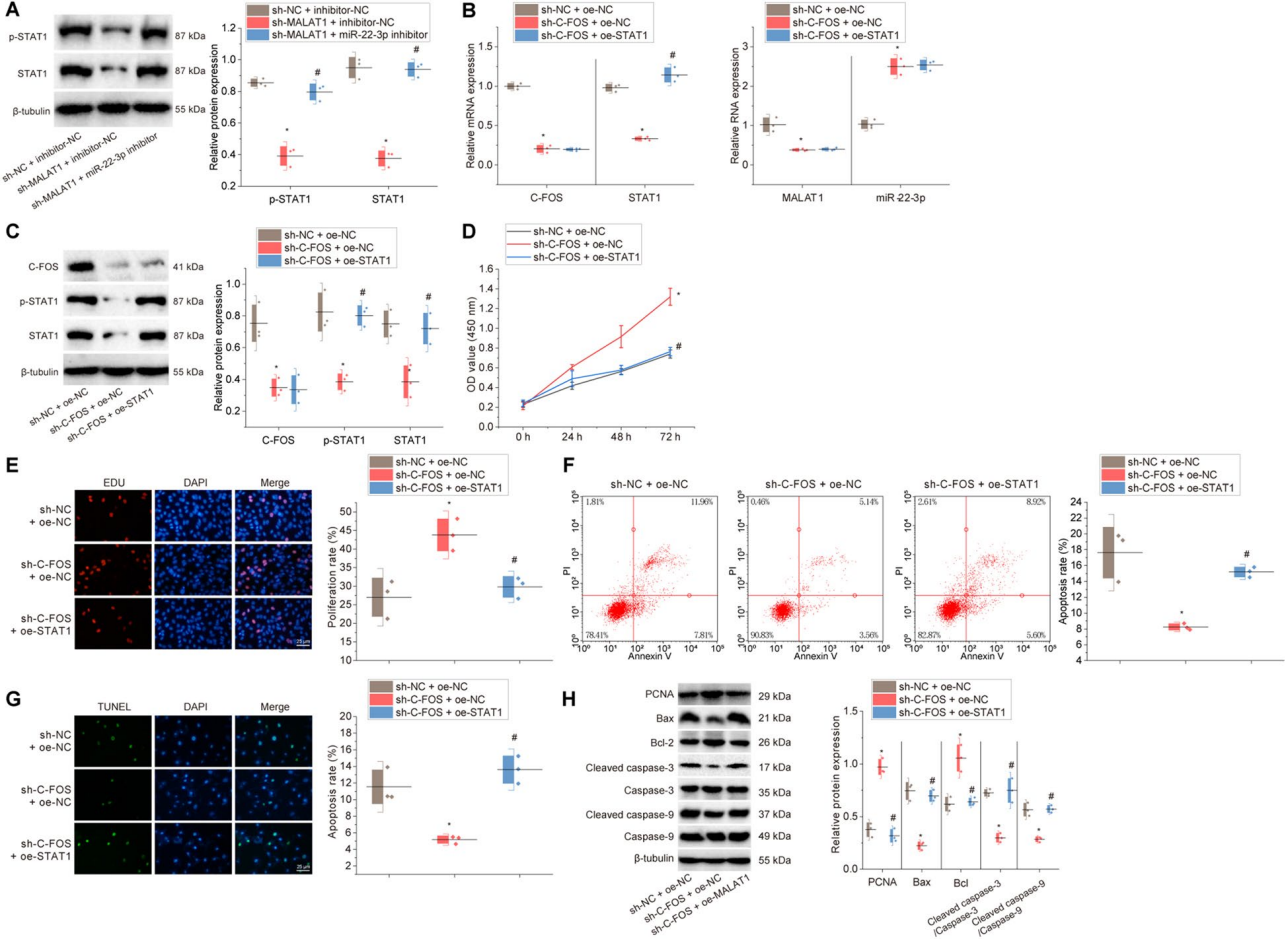
Effects of c-fos on proliferation and apoptosis of KGN cells through regulation of the MALAT1/miR-22-3p/STAT1 ceRNA network. **A** Western blot for STAT1 protein expression and STAT1 phosphorylation level in cells treated with sh-MALAT1 alone or combined with miR-22-3p inhibitor, and statistical results of scale values of the protein bands. * *p* < 0.05 *vs.* cells treated with sh-NC + inhibitor-NC; # *p* < 0.05 *vs.* cells treated with sh-MALAT1 + inhibitor-NC. Cells were then treated with sh-c-Fos alone or combined with oe-STAT1. **B**, **C** RT-qPCR and Western blot for c-fos, STAT1, MALAT1 and miR-22-3p expression and STAT1 phosphorylation level and statistical results of scale values of the protein bands; **D** CCK-8 assay for cell viability; **E** EdU assay for cell proliferation and statistical results of proliferation rate; **F** Flow cytometry for apoptosis and statistical results of apoptosis rate; **G** TUNEL staining for apoptosis and statistical results of apoptosis rate; **H** Western blot to detect the expression of proteins related to cell proliferation and apoptosis and statistical results of scale values of the protein bands. In Panels **B**-**H** * *p* < 0.05 *vs.* cells treated with sh-NC + oe-NC; # *p* < 0.05 *vs.* cells treated with sh-c-fos + oe-NC. Cell experiments were repeated three times

Next, we further verified the regulatory roles between MALAT1, miR-22-3p and STAT1 by in vitro cell assays. Western blot results described that in the CTX-treated KGN cells, sh-MALAT1 decreased the STAT1 expression; miR-22-3p inhibitor treatment contributed to increases in STAT1 protein expression and STAT1 phosphorylation level in the CTX-treated KGN cells with MALAT1 knockdown (Fig. 6A). These results suggest that MALAT1 can regulate STAT1 expression by targeting miR-22-3p.

Subsequently, we explored the effect of c-fos on the MALAT1/miR-22-3p/STAT1 axis. RT-qPCR and Western blot assays demonstrated that in CTX-treated KGN cells, sh-c-fos resulted in marked declines in the expression of c-fos, MALAT1 and the phosphorylation level of STAT1 as well as an increase in miR-22-3p expression; additional treatment with oe-MALAT1 elevated the phosphorylation level of STAT1 but failed to alter the expression of c-fos, MALAT1 and miR-22-3p (Fig. 6B-C). Thus, c-fos can affect the phosphorylation level of STAT1 and STAT1 expression by modulating the MALAT1/miR-22-3p axis.

Next, it was further investigated whether c-fos could affect the proliferation and apoptosis of KGN cells by regulating the MALAT1/miR-22-3p/STAT1 ceRNA network. Functional assays elucidated that in CTX-treated KGN cells, cell viability and proliferation were enhanced after knockdown of c-fos, while STAT1 overexpression could reverse the effects (Fig. 6D-E). Flow cytometry and TUNEL assays showed that in the CTX-treated KGN cells, apoptosis was reduced by c-fos knockdown, which could be increased in the presence of STAT1 overexpression (Fig. 6F-G).

In addition, Western blot results also depicted that in the CTX-treated KGN cells, the expression of PCNA and Bcl-2 was increased while that of Bax, Cleaved caspase-3 and Cleaved caspase-9 decreased in response to c-fos knockdown; these changes could be reversed by additional overexpression of STAT1 (Fig. 6H).

### The c-fos-mediated MALAT1/miR-22-3p/STAT1 ceRNA network exacerbates ovarian function impairment in POI mice

**Fig. 7:**
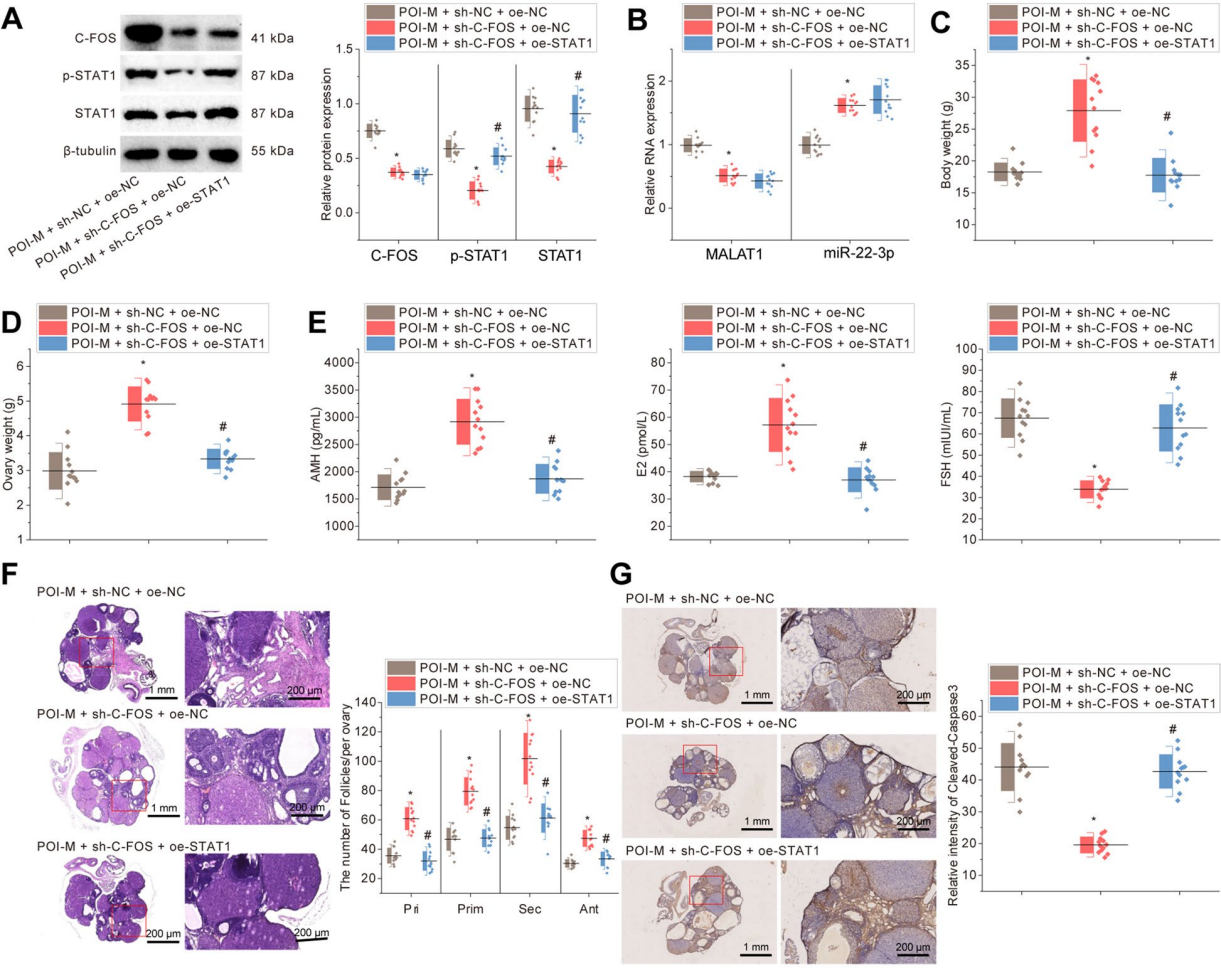
Effects of knockdown of c-fos or overexpression of STAT1 on POI mice. Note: POI mice were treated with sh-c-Fos alone or combined with oe-STAT1. **A** Western blot detection of c-fos and STAT1 protein expression and STAT1 phosphorylation level in ovarian tissues of mice and statistical results of scale values of the protein bands; **B** RT-qPCR detection of MALAT1 and miR-22-3p expression in ovarian tissues of mice; **C** Detection of body weight of mice; **D** Detection of ovarian weight of mice; **E** ELISA of serum AMH, E2 and FSH levels in mice; **F** H&E staining of ovarian tissue structure in mice and statistical results of the number of follicles in each ovary; **G** Cleaved-Caspase3 immunohistochemistry of ovarian apoptosis in mice and statistical results of relative intensity of positive staining. In Panels **A**-**G** * *p* < 0.05 *vs.* POI mice treated with sh-NC + oe-NC; # *p* < 0.05 *vs.* POI mice treated with sh-c-fos + oe-NC. *n* = 12

To detect the effect of c-fos/MALAT1/miR-22-3p/STAT1 axis on ovarian function in POI mice, we verified it by adenovirus injection. Based on RT-qPCR and Western blot results (Fig. 7A-B), sh-c-fos treatment contributed to decreased c-fos and MALAT1 expression, increased miR-22-3p expression, and diminished STAT1 protein expression and STAT1 phosphorylation level. oe-MALAT1 failed to change miR-22-3p, c-fos and MALAT1 expression but notably increased the protein expression and phosphorylation level of STAT1 in sh-c-fos-treated POI mice.

Moreover, both body weight and ovarian weight of POI mice were increased by c-fos knockdown, which could be decreased by STAT1 overexpression (Fig. 7C-D). In addition, ELISA results showed that AMH and E2 levels were higher and FSH expression was lower in the presence of c-fos knockdown, while STAT1 overexpression counteracted these effects (Fig. 7E). H&E staining revealed that c-fos knockdown resulted in an increased number of follicles, a more regular granular cell layer and a reduced inflammatory cell infiltration, the trends of which were reversed by STAT1 overexpression (Fig. 7F). As detected by Cleaved-Caspase3 immunohistochemical analysis, there was a decrease in apoptosis in the presence of c-fos knockdown, while STAT1 overexpression increased the apoptosis (Fig. 7G).

## Discussion

Our results have demonstrated that in POI, c-fos can regulate the progression of this disease through the MALAT1/miR-22-3p/STAT1 axis. Specifically, activating the MALAT1/miR-22-3p/STAT1 axis by c-fos could regulate KGN cell behavior and ovarian function in POI mice.

Initially, we observed high expression of c-fos and MALAT1 in both *in viv*o and in vitro models and found that c-fos directly targeted MALAT1 as a positive regulator. Our findings are supported by numerous studies. For example, high expression of MALAT1 can inhibit KGN cell proliferation and aggravate the onset and progression of ovarian diseases [30]. Moreover, low expression of MALAT1 can inhibit cystic follicle disease through inhibition of p53 degradation by binding to MDM2 complexes [31]. c-Fos is upregulated in Rb-deficient preantral follicles, and deficiency of the Rb gene in ovarian granulosa cells can result in POI [28]. JDP2 complex reformed by rapid c-fos degradation is accountable for prevention of premature reproductive senescence in a mouse model [32]. Intriguingly, previous research has shown that MALAT1 can upregulate c-fos expression in trophoblast cells [33] and that MALAT1 can be activated by c-Fos in renal cell carcinoma, providing strong evidence for the targeting relationship between the two. However, the specific interaction between c-Fos and MALAT1 in the context of POF has been rarely reported.

Additionally, we revealed in the current study that MALAT1 directly targeted miR-22-3p as a negative regulator. By comparing differentially expressed miRNAs in the database, we discovered that miR-22-3p had the most significant differential expression in POI, indicating its importance in disease progression. MALAT1 can affect ovarian disease progression by regulating downstream miRNAs such as miR-145-5p [34], miR-1271-5p [35], miR-503-5p [36], and miR-506 [37], suggesting that MALAT1 might affect POI by regulating downstream miRNAs. Of note, in hepatocellular carcinoma, MALAT1 acts as a ceRNA to accelerate the degradation of apoptosis inhibitor protein by targeting miR-22-3p [38], which supports our analysis results.

Furthermore, we found miR-22-3p could target and inhibit STAT1. Another study of human polycystic ovary syndrome found that STAT1 activation promotes KGN cell apoptosis and inhibits proliferation [39]. Moreover, STAT1 can reverse the stimulation of porcine ovarian cell proliferation and inhibition of apoptosis [40]. Besides, the STAT1 pathway is positively regulated by MALAT1 [41]. Interestingly, miR-22* can downregulate the expression of STAT1 in the reproductive tracts from chickens [42]. This provided us with clues and evidence of the regulatory relationship between miR-22-3p and STAT1. In our study, it was demonstrated that the c-fos-mediated MALAT1/miR-22-3p/STAT1 ceRNA network inhibited proliferation and promoted apoptosis of KGN cells in vitro and exacerbated ovarian function impairment in POI mice.

## Conclusion

**Fig. 8:**
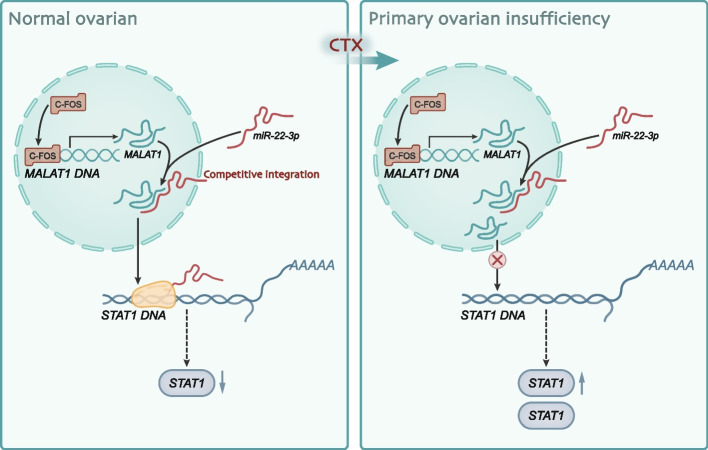
Molecular mechanism of the c-fos-regulated MALAT1/miR-22-3p/STAT1 ceRNA network involved in POI development. Note: c-fos expression is increased in POI cells, and c-fos binds to the MALAT1 promoter region and promotes MALAT1 expression. MALAT1 acts as a ceRNA that competitively binds to miR-22-3p, reducing the targeting inhibitory effect of miR-22-3p on STAT1 and promoting STAT1 expression, ultimately leading to POI deterioration

Based on our results, we can conclude that c-fos is a direct positive regulator of MALAT1 and that MALAT1 is a direct negative regulator of miR-22-3p. c-fos may target and suppress STAT1 expression by upregulating MALAT1 expression while competitively binding with miR-22-3p, ultimately leading to the occurrence of POI (Fig. 8). Our research may provide a new theoretical basis and molecular targets for the diagnosis and treatment of POI. However, there are still several limitations in this study. Firstly, the results were only validated in the in vitro cell experiments and in vivo animal experiments, and further verification is warranted in clinical samples. Secondly, the number of transcription factors studied in this article is relatively small, and transcriptome sequencing in future work may provide a deeper exploration of the pathogenic mechanism of POI.

## Supplementary Information

**Additional file 1: Figure S1.** Flow chart for constructing a POI mouse model* in vivo*.

**Additional file 2: Figure S2.** Effects of c-fos on apoptosis and proliferation of KGN cells. Note: A-B: Cell proliferation after knockdown or overexpression of c-fos detected by EdU assay and statistical results of proliferation rate; C-D: TUNEL staining to detect cell apoptosis after knockdown or overexpression of c-fos and statistical results of apoptosis rate; E-F: Flow cytometry detection of cell apoptosis after knockdown or overexpression of c-fos and statistical results of apoptosis rate. In Panels A-F, * *p* < 0.05* vs.* cells treated with sh-NC or oe-NC. Cell experiments were repeated three times.

**Additional file 3: Table S1.** RT-qPCR primer sequences.

**Additional file 4: Table S2.** Western blot antibody information.

**Additional file 5: Table S3.** Vector sequences for luciferase activity assay.

## Data Availability

The data underlying this article will be shared on reasonable request to the corresponding author.
