# Supplementary material for: Transcription factor c-fos induces the development of premature ovarian insufficiency by regulating MALAT1/miR-22-3p/STAT1 network
Source: J Ovarian Res. 2023 Jul 21;16:144. doi: 10.1186/s13048-023-01212-3 (PMC10362627; doi:10.1186/s13048-023-01212-3)
Supplement: Supplementary file 3 — Additional file 3: Table S1. RT-qPCR primer sequences. [file 13048_2023_1212_MOESM3_ESM.docx]

**Table S1. RT-qPCR primer sequences**

| Gene | Sequences |
| --- | --- |
| c-Fos (mouse) | Forward: 5'-GGTGAAGACCGTGTCAGGAG-3'  Reverse: 5'-TATTCCGTTCCCTTCGGATT-3' |
| c-Fos (human) | Forward: 5'-TGTCTGTGGCTTCCCTTGAT-3'  Reverse: 5'-ATCAAAGGGCTCGGTCTTCA-3' |
| MALAT1 (mouse) | Forward: 5'-GGGGGAATGGGGGCAAAATA-3'  Reverse: 5'-AACTACCAGCAATTCCGCCA-3' |
| MALAT1 (human) | Forward: 5'-TCCTAAGGTCAAGAGAAGTGTCAG-3'  Reverse: 5'-GTGGCGATGTGGCAGAGAA-3' |
| GAPDH (human) | Forward: 5'-CTCTGCCCCCTCTGCTGAT-3'  Reverse: 5'-GTGCAGGAGGCATTGCTGAT-3' |
| GAPDH (mouse) | Forward: 5'-CCTTGAGATCAACACGTACCAG-3'  Reverse: 5'-CGCCTGTACACTCCACCAC-3' |
| miR-22-3p (human) | Forward: 5'-AAGCTGCCAGTTGAAGAACTGT-3'  Reverse: Reverse universal primer |
| U6 | Forward: 5'-CTCGCTTCGGCAGCACA-3'  Reverse: 5'-AACGCTTCACGAATTTGCGT-3' |
