# Supplementary material for: Transcription factor c-fos induces the development of premature ovarian insufficiency by regulating MALAT1/miR-22-3p/STAT1 network
Source: J Ovarian Res. 2023 Jul 21;16:144. doi: 10.1186/s13048-023-01212-3 (PMC10362627; doi:10.1186/s13048-023-01212-3)
Supplement: Supplementary file 4 — Additional file 4: Table S2. Western blot antibody information. [file 13048_2023_1212_MOESM4_ESM.docx]

**Table S2. Western blot antibody information**

| Name | Product No. | Dilution ratio | Brand | Country |
| --- | --- | --- | --- | --- |
| Beta-tubulin | ab6046 | 1: 5000 | Abcam | UK |
| c-Fos | ab27793 | 1:1000 | Abcam | UK |
| STAT1 | ab47425 | 1:1000 | Abcam | UK |
| p-STAT1 | ab109461 | 1:1000 | Abcam | UK |
| Cleaved caspase-3 | ab32042 | 1:1000 | Abcam | UK |
| Cleaved caspase-9 | #9509 | 1:1000 | CST | USA |
| Caspase-3 | ab32499 | 1:1000 | Abcam | UK |
| Caspase-9 | ab18107 | 1:1000 | Abcam | UK |
| Bcl-2 | ab32124 | 1:1000 | Abcam | UK |
| Bax | ab32503 | 1:1000 | Abcam | UK |
| PCNA | A0264 | 1:1000 | ABclonal | USA |
