# Supplementary material for: Transcription factor c-fos induces the development of premature ovarian insufficiency by regulating MALAT1/miR-22-3p/STAT1 network
Source: J Ovarian Res. 2023 Jul 21;16:144. doi: 10.1186/s13048-023-01212-3 (PMC10362627; doi:10.1186/s13048-023-01212-3)
Supplement: Supplementary file 5 — Additional file 5: Table S3. Vector sequences for luciferase activity assay. [file 13048_2023_1212_MOESM5_ESM.docx]

**Table S3. Vector sequences for luciferase activity assay**

| Name | Sequences |
| --- | --- |
| MALAT1-WT | 5'-AGCAGAAAACAGCAGGCAGCUG-3' |
| MALAT1-MUT | 5'-AGCAGAAAAGCAFCAAUGACUG-3' |
| STAT1-WT | 5'-UCAGUCUUUUUUCCAGCAGCUC-3' |
| STAT1-MUT | 5'-UCAGUCUUUCCAUACUACC-3' |

Note: WT, wild type; MUT, mutant type.
